# Enhancement of Magnesium Oxysulfate Cement by Acid Modifiers and Its Reaction Mechanism

**DOI:** 10.3390/ma18071432

**Published:** 2025-03-24

**Authors:** Zixuan Qiao, Wenqiang Fan, Yuting Zhang, Xinyu Fu, Hongjian Yang, Fuqiang Zhang

**Affiliations:** 1 School of Chemical Engineering and Technology, Hebei University of Technology, Tianjin 300401, China; 18032151363@163.com (Z.Q.); 13287113703@163.com (Y.Z.); 19871691036@163.com (X.F.); 2 Tianjin Cheng’an Thermal Power Co., Ltd., Tianjin 300299, China; 18629492513@163.com

**Keywords:** magnesium oxysulfate cement, 2-phosphonobutane-1,2,4-tricarboxylic acid, mechanical properties, hydrolyzed polymaleic anhydride, water resistance

## Abstract

To address the challenges of low mechanical strength and poor water resistance in magnesium oxysulfate cement (MOSC), this study explores the incorporation of 2-phosphonobutane-1,2,4-tricarboxylic acid (PBTC) and hydrolyzed polymaleic anhydride (HPMA) as modifiers. Advanced analytical techniques, including X-ray diffraction, scanning electron microscopy, Fourier transform infrared spectroscopy, Raman spectroscopy, and thermogravimetric differential scanning calorimetry, were employed to analyze the physical phase composition and microscopic structure of MOSC hydration products. These analyses provided insights into the enhancement mechanisms associated with PBTC and HPMA. The findings revealed that the chelation of PBTC and HPMA with Mg^2+^ influenced the hydration process of MOSC, prolonged its setting time, and facilitated the emergence of a new needle-and-rod crystalline phase (5·1·7 phase) within the hydration products. This water-insoluble phase, characterized by a three-dimensional network structure of interspersed crystals, contributed to improved mechanical strength and water resistance. When the doping level of HPMA is 2.00%, the 28-day compressive strength of MOSC reached 104.42 MPa, which exceeded that of the control sample by 127.45%. The softening coefficient was as high as 0.94. The results of this study show that PBTC and HPMA, as modifiers, can effectively improve the mechanical properties and water resistance of MOSC. Their influence on the hydration mechanism and crystallization process of MOSC provides a certain theoretical basis for the practical engineering applications and sustainable development of MOSC.

## 1. Introduction

Magnesium oxysulfate cement (MOSC), an innovative green building material comprising lightly burned magnesium oxide (LBM) and a magnesium sulfate solution, exhibits several advantages, such as its light weight, early strength, fire resistance, halogen-free properties, and anti-corrosion. These characteristics have enabled its application in producing fireproof door cores, decorative components, tubes, and architectural elements [1,2].

Despite its advantages, MOSC has certain disadvantages, such as low early mechanical strength and poor water resistance, which restrict its application range. To address these challenges, researchers have been investigating the use of specialized modifiers to improve the mechanical properties and water resistance of MOSC, which will facilitate its wider application in the construction industry. Between 30 °C and 120 °C, the hydration products of MOSC generally consist of Mg(OH)₂ and five phases of xMg (OH)₂·yMgSO₄·zH₂O, namely 5Mg(OH)₂·MgSO₄·3H₂O (5·1·3 phase), 5Mg(OH)₂·Mg SO₄·7H₂O (5·1·7 phase), 3Mg(OH)₂·MgSO₄·8H₂O (3·1·8 phase), Mg(OH)₂·2MgSO₄·3H₂O (1·2·3 phase), and Mg(OH)₂·MgSO₄·5H₂O (1·1·5 phase) [3,4]. Their formation is related to the molar ratios of MgO/MgSO₄ and MgSO₄/H₂O. When the molar ratio of MgO/MgSO₄ is between 3 and 9, it is conducive to the formation of the 3-phase and 5-phase [5]. Usually, the lamellar Mg(OH)₂ is not resistant to water and has low mechanical strength, while the 5·1·7 phase is considered as the main source of strength [6]. In addition to the method of adjusting the above raw material ratios, modifiers can also be used to promote the formation of the strength-related phases. Among the various modifiers, acid modifiers have garnered the most attention. Organic carboxylic acid modifiers, including citric acid [7,8,9], gluconic acid [10,11], and malic acid [12,13], are widely employed, alongside inorganic phosphoric acid and its salt modifiers, such as phosphoric acid [14,15], potassium dihydrogen phosphate, and sodium dihydrogen phosphate [16,17]. In recent years, organophosphonic acid modifiers have emerged as promising candidates for MOSC modification. Examples include aminotrimethylene phosphonic acid, diethylenetriamine pentamethylene forked phosphonic acid, and hydroxyethylene forked diphosphonic acid [18,19]. The modification mechanism of these modifiers primarily involves chemical interactions. Specifically, the addition of modifiers alters the hydration reaction pathway of MOSC. The ligands of the modifiers form organic magnesium complexes by coordinating with [Mg(OH)(H_2_O)_x_]^+^ and subsequently interact with Mg^2+^, SO_4_^2−^, and H_2_O in the system to generate a 5·1·7 phase. This phase is characterized by a crisscrossed, needle-and-rod structure interwoven into a three-dimensional mesh, significantly improving the compactness, mechanical properties, and water resistance of MOSC. Research has highlighted the use of 2-phosphonobutane-1,2,4-tricarboxylic acid (PBTC) [20,21,22,23] and hydrolyzed polymaleic anhydride (HPMA) [24,25] as scale inhibitors in industrial circulating coolant water systems, low-pressure boilers, and analogous applications. Their effectiveness is attributed to their strong chelating abilities with metal ions. Nevertheless, there is limited exploration of these compounds in the context of cement modification, leaving a gap in understanding their potential applications in this domain.

This study aims to improve the mechanical properties and water resistance of MOSC by using PBTC and HPMA as modifiers. The modification effects were assessed through compressive strength, flexural strength, and softening coefficient measurements. Advanced characterization techniques, including X-ray diffraction (XRD), scanning electron microscopy (SEM), Fourier transform infrared spectroscopy (FT-IR), Raman spectroscopy and thermogravimetric differential scanning calorimetry (TG-DSC), were used to examine the physical composition and microstructure of MOSC in detail. This analysis provided insights into its intrinsic properties, structural characteristics, and the underlying modification mechanism.

## 2. Materials and Methods

### 2.1. Raw Materials

**Figure 1:**
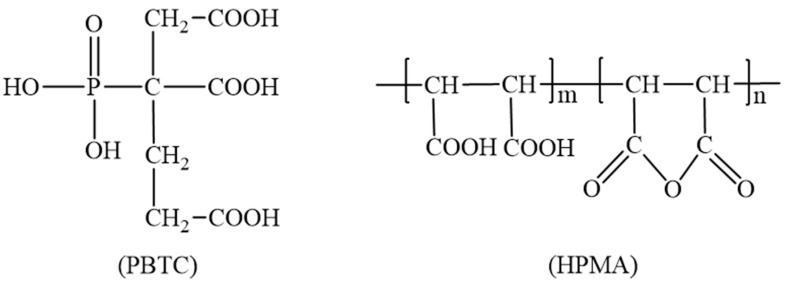
Chemical structural formulas of PBTC and HPMA.

**Figure 2:**
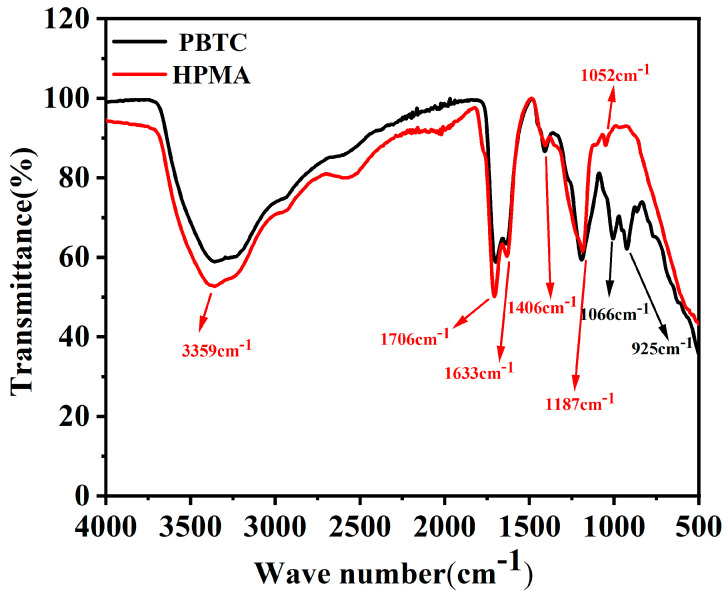
FT-IR spectra of PBTC and HPMA.

The LBM utilized in this experiment was obtained from Huafeng Magnesium Industry Co., Ltd., located in Haicheng City, Liaoning Province, China. The activity of the LBM was determined to be 63.00% using the standard hydrometry method. Its chemical composition was analyzed via X-ray fluorescence, with the results presented in Table 1. Magnesium sulfate heptahydrate was acquired from Yongxing Chemical Co. (Ningbo, China). The PBTC (50% in H_2_O) and HPMA (50% in H_2_O) were purchased from Tianjin Aoberson Biotechnology Co., Ltd. (Tianjin, China). The chemical structures of both compounds are illustrated in Figure 1. The Fourier transform infrared spectroscopy results of these compounds are shown in Figure 2. The absorption peak at 3359 cm^−1^ corresponds to the O-H stretching vibration of the -COOH groups in PBTC and HPMA. The absorption peak around 1700 cm^−1^ corresponds to the C=O stretching vibration of the -COOH groups. The absorption peak around 1633 cm^−1^ corresponds to the C=C stretching vibration. The absorption peak around 1406 cm^−1^ can be attributed to the in-plane bending vibration of the C-O-H in the -COOH groups. The absorption peak around 1187 cm^−1^ corresponds to the C-O stretching vibration. The absorption peak at 1066 cm^−1^ is the P-O stretching vibration of the -PO_3_H_2_ group in PBTC. The absorption peak at 925 cm^−1^ corresponds to the out-of-plane bending vibration of the O-H bond in the -COOH group of PBTC. The absorption peak at 1052 cm^−1^ corresponds to the C-O-C stretching vibration of HPMA. Tap water from the laboratory was used as the mixing water.

### 2.2. Preparation of MOSC

**Figure 3:**
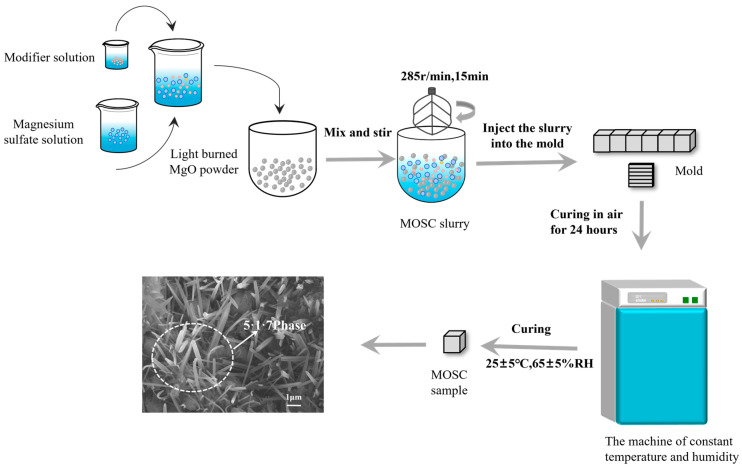
Flow chart of MOSC preparation.

The molar ratio of the experimental raw materials was LBM:MgSO_4_:H_2_O = 11:1:24.92. The dosages of PBTC were 0.00, 0.25, 0.50, 0.75, 1.00, and 1.25 wt%, while the dosages of HPMA were 0.00, 0.50, 1.00, 1.50, 2.00, and 2.50 wt% based on the mass of reactive magnesium oxide, as shown in Table 2. The preparation process for MOSC is illustrated in Figure 3. Initially, the required mass of LBM powder, magnesium sulfate solution, and modifier were weighed. The corresponding dose of the modifier was dissolved in the magnesium sulfate solution. The raw materials were thoroughly mixed to form the MOSC slurry, which was subsequently mixed in a cement paste mixer at 285 r/min for 15 min. The slurry was then poured into molds with dimensions of 40 × 40 × 40 mm^3^ and 10 × 10 × 60 mm^3^, respectively. After curing for 24 h, the samples were transferred into a constant temperature and humidity machine with a temperature of 25 ± 5 °C and a relative humidity of 65 ± 5%. The samples were cured in these conditions until the specified ages before being taken out for testing.

### 2.3. Testing Methods and Characterization

#### 2.3.1. Testing Methods

In this experiment, the samples of MOSC were cured in an environment with a temperature of 25 ± 5 °C and a relative humidity of 65 ± 5%. Compressive strength and flexural strength tests were carried out after 1-day, 3-day, 7-day, 14-day, and 28-day curing periods, respectively, to evaluate mechanical properties. The final mechanical strength data were all the averages of three parallel subsamples. These tests were conducted on an electronic universal testing machine (SANS-UTM5303, Shenzhen Suns Technology Stock Co., Ltd., Shenzhen, China) in line with the standards of GB/T 17671-2021 [26]. The loading rate for the compressive strength test was 2400 N/s, and that for the flexural strength test was 50 N/s. The dimensions of the test samples were 40 × 40 × 40 mm^3^ and 10 × 10 × 60 mm^3^ respectively. The water resistance of the MOSC samples was assessed using the softening coefficient and immersion residual strength. The softening coefficient was calculated using Equation (1), where $R_{\left(w,7\right)}$ represents the compressive strength of the MOSC samples after 28 days of curing followed by 7 days of immersion, and $R_{\left(a,28\right)}$ denotes the compressive strength of the MOSC samples after 28 days of curing.

(1) $$R_{f}=\frac{R_{\left(w,7\right)}}{R_{\left(a,28\right)}}$$

The fluidity of MOSC was tested according to the measurement standard of GB/T 8077-2012 [27]. The MOSC slurry was poured into a frustum-shaped circular mold with dimensions of 36 mm × 60 mm × 60 mm, and then scraped flat. After that, the frustum-shaped circular mold was vertically lifted, and the average of the two maximum diameters in the vertical direction of the slurry after 30 s was taken as the fluidity of the MOSC. The initial and final setting times of MOSC were determined using a Vicat apparatus, following measurement standard GB/T 1346-2011 [28]. The Vicat apparatus was zero-adjusted. The slurry was poured into a frustum-shaped circular mold with dimensions of 65 mm × 70 mm × 40 mm, scraped flat, and then placed in a constant temperature and humidity machine with a temperature of 25 ± 5 °C and a relative humidity of 65 ± 5% to be cured. It was taken out and tested every 30 min. When the initial setting time was approaching, it was tested every 5 min. The initial setting was reached when the change value of the Vicat apparatus pointer indication was 4 ± 1 mm, and this time was recorded as the initial setting time of MOSC. After the initial setting time test was completed, the mold and the slurry were turned together 180° and placed back in the constant temperature and humidity machine. When the final setting time was approaching, it was tested every 15 min. The final setting was reached when obvious marks were no longer left on the surface of the slurry by the pointer, and this time was recorded as the final setting time of MOSC.

#### 2.3.2. Characterization

The MOSC samples cured for 28 days were crushed, and the central portion was ground into powder using an onyx mortar and pestle until it passed through a 75 μm sieve for further characterization. FT-IR spectroscopy (Thermo Fisher Scientific Nicolet iS20, Waltham, MA, USA) was conducted over a wave number range of 500 to 4000 cm^−1^. Raman spectroscopy (Horiba LabRAM HR Evolution, Kyoto, Japan) was performed over a wave number range of 50 to 4000 cm^−1^, with a laser wavelength of 532 cm^−1^. The thermal properties and weight loss of the MOSC samples were evaluated using a thermal analysis system (HCT-3) by heating the powder from room temperature to 1010 °C in a N_2_ atmosphere at a rate of 10 °C/min. The physical phase composition of the MOSC was analyzed using XRD (D8 FOCUS Lynxeye detector, Bruker, Billerica, MA, USA) with a scanning range of 5° to 90°, a step size of 0.019°, and a scanning rate of 0.2 s per step. For microscopic examination, the MOSC samples were broken into approximately 7 × 7 mm blocks, coated with gold, and observed under an SEM (TESCAN MIRA LMS, Brno, Czech Republic) at an operating voltage of 10 kV.

## 3. Results

### 3.1. Compressive Strength

**Figure 4:**
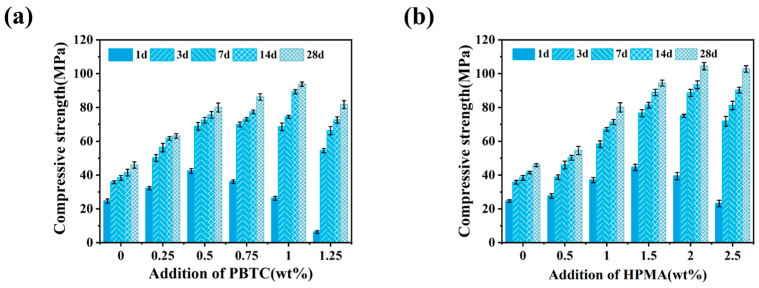
Effect of modifier dosage on the compressive strength of MOSC: (**a**) PBTC; (**b**) HPMA.

The dimensions of the compressive strength test samples are 40 × 40 × 40 mm^3^. Figure 4 illustrates the effect of modifier dosage on the compressive strength of MOSC. Before the PBTC dosage reaches 1% and the HPMA dosage reaches 2%, the compressive strength of MOSC shows an upward trend with the increase in the additive dosage. However, when the PBTC dosage is 1.25% and the HPMA dosage is 2.5%, the compressive strength of MOSC slightly decreases. Additionally, the compressive strength of MOSC at each dosage gradually increased during the 28-day curing period. As shown in Figure 4a, the compressive strengths of the MOSC samples after 28 days of curing were 45.91, 63.13, 79.96, 83.26, 93.80, and 81.71 MPa. When a PBTC dosage of 1.00% was used, the compressive strength of MOSC samples cured for 28 days was 104.31% higher than that of the control group. Furthermore, the 7-day compressive strength of MOSC samples doped with 0.25% and 0.50% PBTC reached 89.07% and 91.14% of their respective 28-day compressive strengths. This indicates that lower PBTC dosages impart early strength characteristics to MOSC. Figure 4b reveals that the compressive strength of MOSC samples after 28 days of curing were 45.91, 54.49, 80.05, 94.46, 104.42, and 102.81 MPa. At an HPMA doping level of 2.00%, the compressive strength of MOSC samples cured for 28 days was 127.45% higher than that of the control group.

### 3.2. Flexural Strength

**Figure 5:**
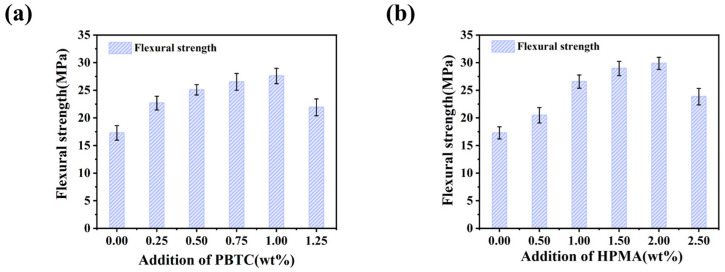
Effect of modifier dosage on the flexural strength of MOSC after 28 days of curing: (**a**) PBTC; (**b**) HPMA.

The dimensions of the flexural strength test samples were 10 × 10 × 60 mm^3^. Figure 5 illustrates the effect of modifier dosage on the flexural strength of MOSC after 28 days of curing. The variation trend of the flexural strength of MOSC with the increase in the additive dosage is similar to that of the compressive strength. As shown in Figure 5a, the flexural strength of the MOSC samples cured for 28 days with a PBTC dosage of 1.00% is 27.59 MPa, which is 59.57% higher than the 17.29 MPa value in the control sample. Similarly, Figure 5b indicates that the flexural strength of the MOSC samples cured for 28 days with a 2.00% HPMA dosage is 29.87 MPa, which is 72.76% higher than the 17.29 MPa of the control sample.

### 3.3. Water Resistance

**Figure 6:**
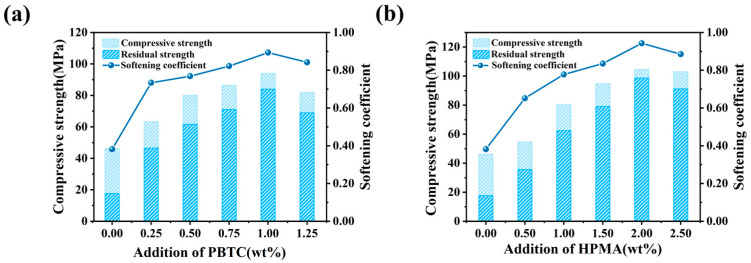
Effect of modifier dosage on the water resistance of MOSC: (**a**) PBTC; (**b**) HPMA.

The water resistance of MOSC was evaluated indirectly through its softening coefficient and immersion residual strength. Figure 6 illustrates the effect of modifier dosage on these parameters. The variation trends of both the softening coefficient and immersion residual strength of MOSC are also similar to that of the compressive strength. The control sample showed a softening coefficient of 0.38 and an immersion residual strength of 17.50 MPa. As depicted in Figure 6a, MOSC with 1.00% PBTC had an immersion residual strength of 80.80 MPa and a softening coefficient of 0.89, which is 134.21% higher than the control. Similarly, Figure 6b shows that MOSC with 2.00% HPMA achieved an immersion residual strength of 94.44 MPa and a softening coefficient of 0.94, which is 147.37% higher than the control. Overall, the water resistance of MOSC was greatly improved by adding both modifiers, and the optimum softening coefficients of both modifiers were around 0.90, which is suitable for the actual production of MOSC.

### 3.4. Fluidity and Setting Time

**Figure 7:**
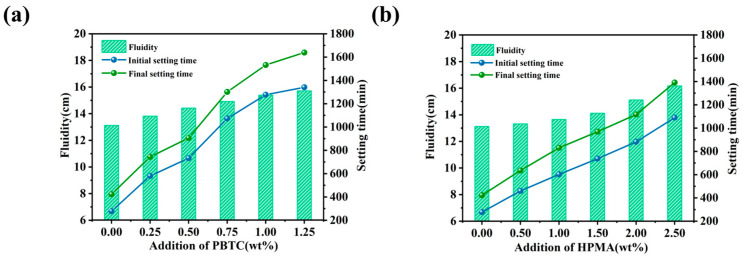
Effect of modifier dosage on the fluidity and setting time of MOSC: (**a**) PBTC; (**b**) HPMA.

Figure 7 illustrates the effect of modifier dosage on the fluidity and setting time of MOSC. As shown in Figure 7a, the fluidity of the MOSC slurry increases with the increasing PBTC dosage. The control sample exhibited a fluidity of 13.10 cm, while MOSC with 1.25% PBTC showed a fluidity of 15.70 cm. The incorporation of PBTC significantly prolonged the setting time of MOSC. For the control sample, the initial and final setting time were 278 min and 423 min, respectively. However, MOSC with 1.25% of PBTC had an initial setting time of 1341 min and a final setting time of 1639 min. A similar trend was observed in Figure 7b, where the incorporation of HPMA increased the fluidity and setting time of MOSC. The fluidity at 2.00% HPMA incorporation was 16.15 cm, with the initial and final setting time of 1089 and 1391 min, respectively. The addition of both modifiers greatly extended the setting time of MOSC, possibly due to their influence on the hydration reaction pathway. Both PBTC and HPMA likely chelated with [Mg(OH)(H_2_O)_x_]^+^ during the hydration reaction, covering the surface of MgO particles that are not yet involved in hydration and retarding the hydrolysis process. As a result, the overall hydration reaction rate of MOSC decreased, thereby prolonging the setting time [29].

### 3.5. FT-IR and Raman Analysis

**Figure 8:**
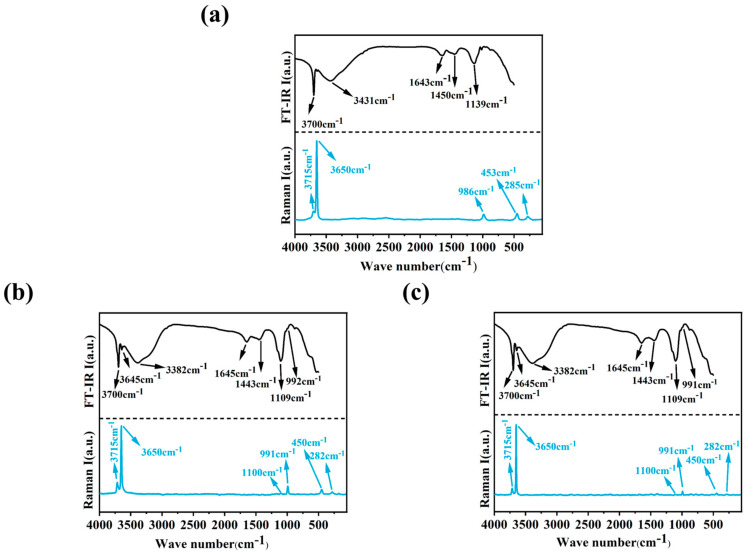
FT-IR and Raman spectra of MOSC sample: (**a**) sample C; (**b**) sample P4; (**c**) sample H4.

Figure 8 presents the FT-IR and Raman spectra of the MOSC samples, which characterize the functional group composition of their hydration products. The peak at 3700 cm^−1^ corresponds to the O-H bond stretching vibration in the hydration product Mg(OH)_2_, while the peak at 3645 cm^−1^ is attributed to the O-H stretching vibration of the water ligand. The intensity of this peak is significantly enhanced in the system with added modifiers, likely because the modifier ligands react with [Mg(OH)(H_2_O)_x_]^+^ to form an organomagnesium chelate. This chelate further interacts with Mg^2+^, SO_4_^2−^, and OH^−^ in solution, promoting the formation of the 5·1·7 phase. Peaks at 3382 cm^−1^ and 1643 cm^−1^ are associated with the O-H stretching and bending vibrations of bound water [30]. The peak around 1450 cm^−1^ corresponds to the O-C-O stretching vibrations of CO_3_^2−^, and characteristic absorption peaks for the ν_3_(SO_4_), ν_1_(SO_4_), and ν_2_(SO_4_) stretching vibrations of SO_4_^2−^ appear at 1139 cm^−1^, 991 cm^−1^, and 450 cm^−1^, respectively [18,31].

### 3.6. TG-DSC Analysis

**Figure 9:**
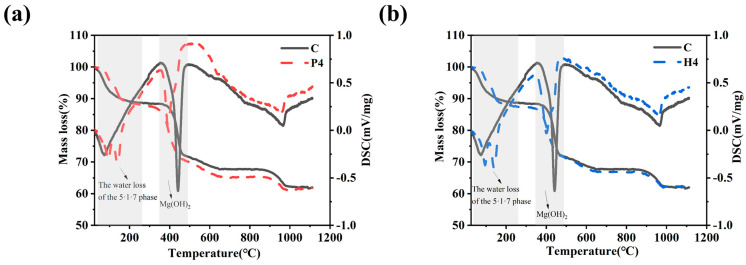
TG-DSC of MOSC sample: (**a**) samples C and P4; (**b**) samples C and H4.

Figure 9 presents the weight loss curve from the TG-DSC analysis of the MOSC sample, with the corresponding heat absorption peaks indicating the presence of MOSC hydration products. The P4 and H4 samples exhibited five distinct weight loss stages between 25 °C and 1010 °C, and two weight loss stages between 25 °C and 270 °C, which correspond to the removal of seven crystalline water molecules [32,33]. This decomposition follows Equation (2):

(2) $$5\mathrm{Mg}{\left(\mathrm{OH}\right)}_{2}\cdot {\mathrm{MgSO}}_{4}\cdot 7H_{2}O\left(s\right)\to \;5\mathrm{Mg}{\left(\mathrm{OH}\right)}_{2}\cdot {\mathrm{MgSO}}_{4}\left(s\right)+7H_{2}O\left(g\right)$$

The weight losses were 12.12% and 12.52% for the P4 and H4 samples, respectively, compared to 11.55% in the control group, indicating that the addition of PBTC and HPMA promotes the formation of 5Mg(OH)_2_·MgSO_4_·7H_2_O. The two weight loss stages observed between 270 °C and 470 °C correspond to the loss of water from 5Mg(OH)_2_·MgSO_4_ and Mg(OH)_2_ [34]. The decomposition reactions are represented by Equations (3) and (4):

(3) $$5\mathrm{Mg}{\left(\mathrm{OH}\right)}_{2}\cdot {\mathrm{MgSO}}_{4}\left(s\right)\to 5\mathrm{MgO}\left(s\right)+{\mathrm{MgSO}}_{4}\left(s\right)+5H_{2}O\left(g\right)$$

(4) $$\mathrm{Mg}{\left(\mathrm{OH}\right)}_{2}\left(s\right)\to \mathrm{MgO}\left(s\right)+H_{2}O\left(g\right)$$

The weight losses were 17.25% and 17.72% for the modified samples, compared to 18.49% for the control group, indicating a reduction in Mg(OH)_2_ production with the addition of the modifier. The decomposition observed between 470 °C and 685 °C corresponds to the breakdown of the 5Mg(OH)_2_·MgSO_4_ residue [35], as represented by Equation (5):

(5) $$5\mathrm{Mg}{\left(\mathrm{OH}\right)}_{2}\cdot {\mathrm{MgSO}}_{4}\left(s\right)\to 5\mathrm{MgO}\left(s\right)+{\mathrm{MgSO}}_{4}\left(s\right)+5H_{2}O\left(g\right)$$

Between 685 and 1010 °C, the decomposition loss of MgCO_3_ and MgSO_4_ corresponds with the decomposition Equation (6) and Equation (7), respectively:

(6) $${\mathrm{MgCO}}_{3}\left(s\right)\to \mathrm{MgO}\left(s\right)+{\mathrm{CO}}_{2}\left(g\right)$$

(7) $${\mathrm{MgSO}}_{4}\left(s\right)\to \mathrm{MgO}\left(s\right)+{\mathrm{SO}}_{3}\left(g\right)$$

### 3.7. XRD Analysis

**Figure 10:**
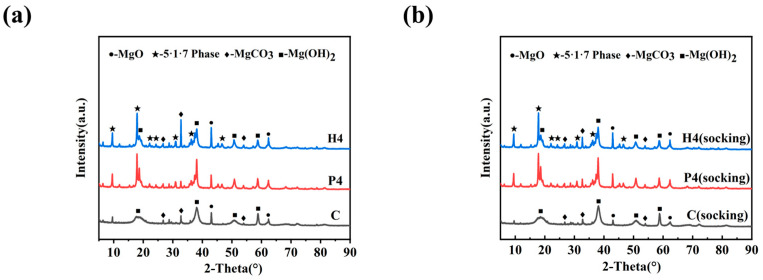
Physical phase composition of MOSC before and after water immersion: (**a**) pre-immersion; (**b**) after immersion.

Figure 10 shows the effect of modifier dosing on the physical phase composition of MOSC before and after water immersion. As seen in Figure 10a, the physical phase compositions of the control group C after 28 days of conservation are MgO, MgCO_3_, and Mg(OH)_2_ phases. A new 5·1·7 phase appeared in the experimental groups P4 and H4, which were doped with the modifier based on the above three phases, which provided a source of improvement in the mechanical properties and water resistance properties of MOSC [35,36]. Figure 10b reveals that there is almost no difference in the phase composition of the samples before and after immersion. Notably, the 5·1·7 phase in the experimental group remains intact, with the intensity of its diffraction peaks comparable to those observed before immersion. This result indicates that the 5·1·7 phase is insoluble in water and exhibits excellent water resistance. This characteristic is further evidenced by the increased softening coefficients observed in the control groups P4 and H4.

### 3.8. SEM and EDS Analysis

**Figure 11:**
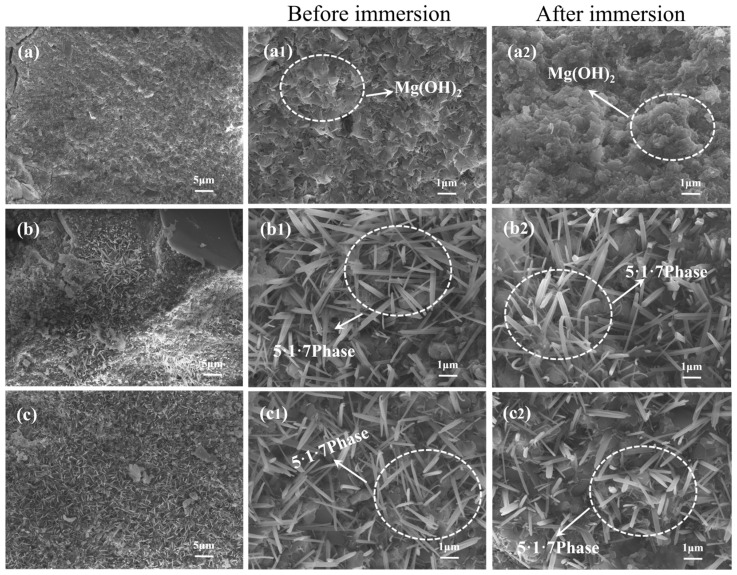
Microscopic morphology of MOSC sample before and after immersion in water: (**a**) sample C; (**b**) sample P4; (**c**) sample H4.

**Figure 12:**
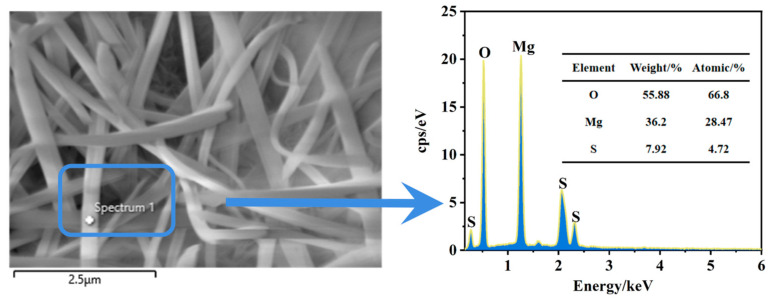
Energy-dispersive spectroscopy spectrum of 5·1·7 phase.

Figure 11 presents the microscopic morphology of MOSC before and after water immersion. In Figure 11(a1,a2), the morphology of control group C prior to immersion primarily consists of loose, laminar Mg(OH)_2_ flakes with relatively sharp edges. However, after immersion, some Mg(OH)_2_ appears partially dissolved, with the initially sharp angles becoming rounded, indicating the insufficient water resistance of Mg(OH)_2_. In Figure 11(b1,b2), it is evident that the addition of PBTC introduces a dense needle-and-rod phase in the MOSC system. According to the EDS analysis in Figure 12, this phase is identified as the 5·1·7 phase [18]. Notably, after immersion in water, the 5·1·7 phase does not exhibit dissolution, demonstrating stability consistent with the XRD analysis. The 5·1·7 phase substantially enhances the softening coefficient and mechanical strength of MOSC, a phenomenon also observed in group (c) in Figure 11.

## 4. Discussion

The hydration reaction process of the MOSC can be divided into five distinct stages. The first stage is represented by Equation (8):

(8) $$\mathrm{MgO}\left(s\right)+\left(x+1\right)H_{2}O\to {\left[\mathrm{Mg}\left(\mathrm{OH}\right){\left(H_{2}O\right)}_{x}\right]}^{+}+{\mathrm{OH}}^{-}$$

In this first stage, MgO is hydrolyzed in the MgSO_4_ solution, producing [Mg(OH)(H_2_O)_x_]^+^ and OH^−^, which increase the system’s alkalinity. In the absence of any added modifier, the second stage proceeds as shown in Equation (9):

(9) $${\left[\mathrm{Mg}\left(\mathrm{OH}\right){\left(H_{2}O\right)}_{x}\right]}^{+}+{\mathrm{OH}}^{-}\to \mathrm{Mg}{\left(\mathrm{OH}\right)}_{2}\left(s\right)+{\mathrm{xH}}_{2}O$$

**Figure 13:**
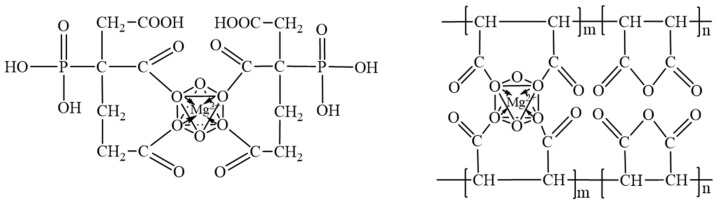
Chelates of PBTC, HPMA, and Mg^2+^ octahedral bodies.

**Figure 14:**
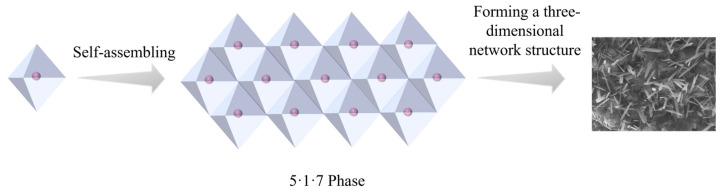
The formation of the 5·1·7 phase.

**Figure 15:**
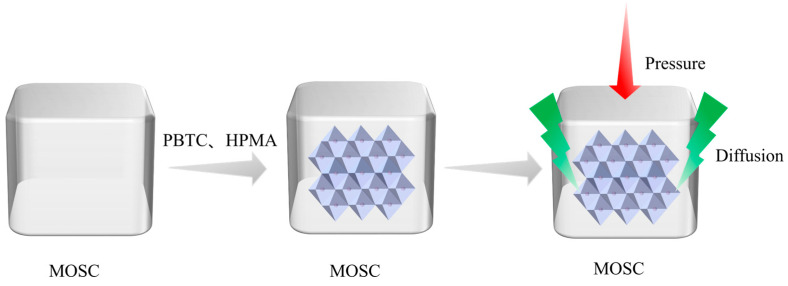
The enhancement mechanism of PBTC and HPMA on magnesium oxysulfate cement.

The [Mg(OH)(H_2_O)_x_]^+^ is unstable and readily reacts with OH^−^ in alkaline environments, forming the lamellar weak phase Mg(OH)_2_. The third stage occurs with the addition of a modifier, and the modifier used in this paper, the organophosphonic acid PBTC, possesses two -COOH and one -C-PO(OH)_2_ group, which, upon dissociation, can form a 1:1 or 1:2 type of chelate with [Mg(OH)(H_2_O)_x_]^+^ [19,37,38]. As shown in Figure 13, each monomer of the hydrolyzed polymaleic anhydride (HPMA) contains two -COOH groups, which dissociate to form COO^−^ with a negative charge in an alkaline environment. This allows HPMA to readily chelate with [Mg(OH)(H_2_O)_x_]^+^, forming a stable six-membered chelate [39,40]. After formation, this chelate attaches to the surfaces of unhydrated MgO particles, delaying the first stage of the hydration reaction. In this process, HPMA preferentially consumes [Mg(OH)(H_2_O)_x_]^+^, thereby reducing the formation of Mg(OH)_2_. As hydration progresses, the chelate reacts with SO_4_^2−^, Mg^2+^, and OH^−^ in solution to form 5·1·7 nuclei, which continue to grow into the 5·1·7 phase [13,41], as shown in Figure 14. In the fourth stage, the concentration of reactants decreases with the generation of products, and the hydration reaction rate of MOSC decreases; in the fifth stage, the hydration products are gradually generated, and the hydration reaction rate tends to stabilize [42,43]. The needle-and-rod 5·1·7 phase is crisscrossed and tightly stacked in clusters, and its presence prevents the further generation of the weak phase Mg(OH)_2_, which is not water-resistant and has low strength, and is the largest source of strength support for the modified MOSC, as shown in Figure 15.

## 5. Conclusions

In this study, the effects of the acid modifiers PBTC and HPMA on the compressive and flexural strength, softening coefficient, fluidity, setting time, physical phase composition, and microstructure of MOSC were investigated, and the reaction mechanism was discussed. The specific conclusions drawn are as follows:

(1) PBTC and HPMA improved the mechanical properties of MOSC. At a PBTC doping level of 0.50%, the 7-day compressive strength of MOSC reached 91.14% of its 28-day compressive strength, demonstrating early strength development. With 2.00% HPMA doping, the 28-day compressive and flexural strengths of MOSC were 104.42 and 29.87 MPa, respectively, representing increases of 127.45 and 72.76% compared to the control sample.
(2) The incorporation of PBTC and HPMA notably improved the water resistance of MOSC. At 2.00% HPMA doping, the softening coefficient reached a peak value of 0.94, which was 147.37% higher than that of the control sample. Additionally, the modifiers increased the fluidity and prolonged the setting time of MOSC.
(3) The chelating action of PBTC and HPMA produced a new strength phase, the 5·1·7 phase, in the MOSC system. This phase altered the hydration products of MOSC and prevented the formation of Mg(OH)_2_. The needle-and-rod-shaped, cluster-like 5·1·7 phase provides a source of strength to MOSC that can effectively prevent the ingress of free water, thereby improving the mechanical properties and water resistance of MOSC.

## Figures and Tables

**Table 1:** Chemical composition of LBM.

Composition	MgO	SiO_2_	Al_2_O_3_	Fe_2_O_3_	CaO	K_2_O	Others
Mass fraction (%)	85.14	7.05	3.77	0.67	2.99	0.03	3.34

**Table 2:** Modifiers’ blending scheme in MOSC.

Serial Number	C	P1	P2	P3	P4	P5
PBTC (wt%)	0.00	0.25	0.50	0.75	1.00	1.25
Serial number	C	H1	H2	H3	H4	H5
HPMA (wt%)	0.00	0.50	1.00	1.50	2.00	2.50

## Data Availability

The raw data supporting the conclusions of this article will be made available by the authors on request due to privacy.
